# Use of fondaparinux in patients with heparin-induced thrombocytopenia on veno-venous extracorporeal membrane oxygenation: A three-patient case series report

**DOI:** 10.3389/fmed.2023.1112770

**Published:** 2023-02-23

**Authors:** Jitka Rychlíčková, Vladimír Šrámek, Pavel Suk

**Affiliations:** ^1^International Clinical Research Center, St. Anne's University Hospital Brno, Brno, Czechia; ^2^Department of Anesthesiology and Intensive Care, Faculty of Medicine, Masaryk University, St. Anne's University Hospital Brno, Brno, Czechia; ^3^Department of Pharmacology, Faculty of Medicine, Masaryk University, Brno, Czechia

**Keywords:** fondaparinux, pharmacokinetics, heparin-induced thrombocytopenia, extracorporeal membrane oxygenation, pharmacoeconomics

## Abstract

Heparin-induced thrombocytopenia is a life-threatening immune-mediated complication of unfractionated heparin therapy. Fondaparinux is a therapeutic alternative, but it has limited evidence for its use in patients on extracorporeal membrane oxygenation (ECMO). We present a series of three adult patients with COVID-19 on ECMO who were diagnosed with heparin-induced thrombocytopenia after 7–12 days of unfractionated heparin treatment and were switched to fondaparinux. Fondaparinux was initiated with an intravenous loading dose of 5 mg, followed by a dose of 2.5 mg subcutaneously every 8–12 h. Dosage was adjusted according to daily measured anti-Xa concentration with a target range of 0.4–0.7 mg/L. The total duration of treatment with fondaparinux and ECMO ranged from 13 to 26 days. One major bleeding episode unrelated to fondaparinux therapy was observed, and the transfusions requirement was also low in all patients. The ECMO circuit was changed once in each patient. This series provides a deep insight into the use of fondaparinux over an extended period of time in patients on ECMO. Based on the presented data, fondaparinux can be considered a reasonable and affordable anticoagulant in patients without a high risk of bleeding.

## 1. Introduction

Extracorporeal membrane oxygenation (ECMO) is a life-saving therapy for severe acute respiratory distress syndrome (ARDS) in COVID-19 patients. The use of an extracorporeal circuit requires contact of blood with artificial materials (cannulas, tubing, pump, and oxygenator membrane). This leads to the activation of platelets and the coagulation cascade. Anticoagulation is necessary for most ECMO patients to suppress this undesirable phenomenon and prevent circuit clotting. Unfractionated heparin (UFH) is the most frequently used drug because it is cheap, has a short half-life and an antidote, and is relatively easy to monitor. Although the optimal target for UFH anticoagulation is not clearly defined, recent Extracorporeal Life Support Organization (ELSO) guidelines recommend a target anti-Xa range of 0.3–0.7 IU/ml (1). Concordantly, data from COVID-19 patients on ECMO show that the most frequent (two-thirds of patients) therapeutic target is 0.3–0.6 or 0.3–0.7 IU/ml (2). This was associated with the following clinical outcomes: 29% of patients had only bleeding events, 16% had only thrombotic events, and 20% had both bleeding and thrombosis.

A decrease in platelet counts is common in ECMO patient group—mainly due to the consumption in the extracorporeal circuit or sepsis. Moreover, heparin-induced thrombocytopenia (HIT) is diagnosed in 0.8–22.2% (mean 3.7%) patients on ECMO (3). Platelet-activating immune complexes have been described in patients with COVID-19 (4); however, the incidence of HIT in critically ill patients with COVID-19 is comparable (5). HIT is a complication influencing morbidity and mortality in critically ill patients (6) and patients with COVID-19 (7). HIT management is based on the immediate discontinuation of heparin and its replacement with non-heparin alternatives—argatroban, bivalirudin, or fondaparinux among parenteral anticoagulants. In the Czech Republic, only fondaparinux is an available and economically acceptable alternative for longer-term administration, although its use in HIT is off-label (8).

To our knowledge, only a few papers describing the administration of fondaparinux in adult patients on ECMO have been published. A cohort of 8 patients on ECMO treated with fondaparinux 2.5 mg per day due to HIT was reported by Loforte et al. (9). Two case reports describe a young female after mitral valve replacement and having fondaparinux 2.5 mg subcutaneously (SC) daily to provide anticoagulation for 5 days on veno-arterial ECMO (10) and a patient with ARDS due to SARS-CoV-2 infection receiving fondaparinux 7.5 mg SC daily during 5 days of veno-venous ECMO (11). Nevertheless, none of these papers provide a more detailed view on achieved plasma concentrations and longer-term administration of fondaparinux in patients on ECMO, as presented in the following description of three patients.

### 1.1. Fondaparinux and its potential interference with ECMO

Fondaparinux is a synthetic anti-Xa inhibitor with a molecular weight of 1,728, highly selective, and with a high affinity to antithrombin. Fondaparinux binding to antithrombin leads to irreversible conformational changes, which considerably increase the factor Xa inhibition rate (12). The high protein binding of fondaparinux determines the low distribution volume (V_D_). In healthy volunteers, the V_D_ ranges from 7.4 to 10.9 L over the dose range of 2–20 mg of intravenous (IV) fondaparinux resp. 10–10.8 L over the dose range of 2–8 mg of SC fondaparinux and 8.2 L for a single fondaparinux SC 2.5 mg dose (12, 13). The partition coefficient logP of fondaparinux is 0.4 (14). Based on these properties, the effect of ECMO on plasma concentrations of fondaparinux/risk of sequestration can be tentatively predicted using the criteria proposed by Ha et al. (15). Given the small V_D_ (<1 L/kg), ECMO may theoretically increase V_D_; however, when comparing the volume of the ECMO circuit in adults (~600 mL) and the average blood volume (~5 L), only a mild increase in V_D_ can be expected without the need for a higher loading dose. There is also a moderate risk of sequestration of fondaparinux on the ECMO circuit, which may translate into the necessity of higher maintenance dosage.

The anticoagulant effect of fondaparinux can be measured using an anti-Xa assay calibrated with fondaparinux (in mg/L). Anti-Xa assay calibrated with LMWH can also be used, however, a dose-dependent bias must be taken into account. Within the therapeutic range, the anti-Xa assay calibrated with LMWH overestimates fondaparinux concentration by ~20% (16). The test validated with LMWH calibrators (in IU/mL) serves only as a rough guide and may be helpful especially for monitoring of accumulation.

### 1.2. Target plasma concentrations and fondaparinux dosing

Anticoagulation therapy is always a balance between thrombosis (i.e., ECMO circuit dysfunction and decreased circuit lifespan) and bleeding complications. Target anti-Xa levels of fondaparinux for therapeutic, prophylactic, and ECMO indications are not established. Mean peak and trough plasma concentrations observed after 2.5 mg per day were 0.39–0.50 mg/L and 0.14–0.19 mg/L, respectively; in therapeutic dosing reflecting body weight, observed mean peaks and troughs were 1.20–1.26 mg/L, and 0.46–0.62 mg/L, respectively (8, 17, 18). Wahby et al. report very similar peak and trough anti-Xa levels as targets for prophylaxis in critically ill patients (0.47 ± 0.2 mg/L and 0.18 ± 0.1 mg/L, respectively) (19). On the other hand, few data are available confirming similar plasma levels of fondaparinux to be effective in venous thromboembolism prophylaxis (20). The correlation between plasma concentration and clinical outcomes is not proven in larger and/or specific populations (17, 21). The above-mentioned values may thus serve only as an approximate guide for dose titration.

In estimating the dosage of fondaparinux in our ECMO patients, we aimed at plasma concentrations of ~0.4–0.7 mg/L as a boundary between prophylactic and therapeutic levels or the lower part of the therapeutic range.

Our calculation of the individual dose was based on the pharmacokinetic data from healthy volunteers because data from the critically ill are generally unavailable. A loading dose (LD) was used to quickly achieve the target plasma concentration (c_target_) set at 0.6 mg/L in a drug with a long biological half-life. We entered the values of 7–11 L for the V_D_ and 17–21 h for the biological half-life (T_1/2_; taking into account its prolongation in impaired kidney function) (18). The maintenance dose was then estimated based on clearance and adjusted according to repeatedly measured anti-Xa levels taken prior to the next dose. We chose to divide the total daily dose into multiple single doses to minimize fluctuations in plasma levels and to prevent potential bleeding around peak concentrations. We did not expect any significant contribution of ECMO to V_D_ or clearance in our adult patients.

The following formulas were used for calculations:


$$\begin{array}{l} \;LD\;=c_{target}\;\times \;V_{D.}\\ \;MD=c_{target\;}\times \;clearance\\ clearance\;=\;\frac{\ln2\;\times V_{D}}{T_{1/2}} \end{array}$$

A single dose was then calculated from the daily maintenance dose, considering the available size of fondaparinux injections (2.5 mg) and the interval at which this dose should be administered. Generally, the loading dose was 5 mg, followed by the maintenance dose of 2.5 mg every 8–12 h for a patient with more than 60 kg of body weight and normal renal functions. Subsequently, the anti-Xa value was checked 0.5–2 h prior to the next dose almost daily. For dose adjustment, the measured anti-Xa level, renal function, platelet count, and clinical signs of bleeding and thrombosis were considered.

Clotting in the extracorporeal circuit is usually challenging to diagnose. Visual inspection of tubing and membrane oxygenator may detect fibrin deposits or thrombi formation. Significant oxygenator thrombosis leads to an increase in the pressure gradient across the oxygenator (ΔP) and is also associated with impaired gas exchange in the oxygenator. Thrombosis in cannulas, tubing, or especially in the blood pump leads to intravascular hemolysis. Free plasma hemoglobin is a standard marker of its severity, with normal values below 0.5 g/L. Since we record ΔP hourly and measure free plasma hemoglobin daily, we used these parameters to assess anticoagulation efficacy. The most common complication of anticoagulation therapy is bleeding.

## 2. Case descriptions

Fondaparinux was used to provide anticoagulation in three critically ill COVID-19 patients with severe ARDS who developed HIT while on veno-venous ECMO. One patient had positive both HIT antibodies and the aggregation test (patient A), while two other patients had negative HIT antibodies and positive aggregation test (patients B and C). HIT diagnosis was confirmed in the National Reference Laboratory (Institute of Hematology and Blood Transfusion, Prague, Czech Republic) for all three patients. Demographic data and time course of HIT diagnosis and fondaparinux and ECMO therapy are summarized in Table 1. If not stated otherwise, day 0 is the date of HIT diagnosis and commencement of fondaparinux therapy. The glomerular filtration rate was calculated according to the CKD Epidemiology Collaboration Group (GFR CKD-EPI) equation based on serum creatinine; all values are calculated for the actual body surface and stated as median [range].

**Table 1:** Basic demographics and time course.

**Patient**	**Age (years)**	**Sex**	**Weight (kg)**	**Height (cm)**	**ABW (kg)**	**BMI (kg/m^2^)**	**ECMO duration = ICU stay (days)**	**HIT diagnosis (days after UFH started)**	**Fondaparinux therapy duration (days)**	**PRBC units during fondaparinux therapy—total (per day)**	**ECMO circuit exchange (days after HIT diagnosis)**
A	63	F	90	165	70	33.1	25	7	18	6 (0.3)	1
B	61	M	90	180	81	27.8	20	7	13	4 (0.3)	8
C	52	M	118	189	97	33.0	37	12	26	5 (0.2)	2

M, male; F, female; ABW, Adjusted Body Weight; BMI, Body Mass Index; ECMO, Extracorporeal Membrane Oxygenation; PRBC, Packed Red Blood Cells; UFH, Unfractionated Heparin; HIT, Heparin-Induced Thrombocytopenia.

### 2.1. Patient A

**Figure 1:**
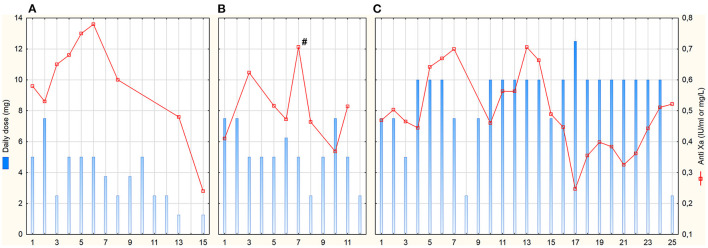
Daily fondaparinux doses and anti-Xa trough levels. Anti-Xa in IU/mL for patient **(A)** (assay calibrated for low molecular-weight heparins), in mg/L for patients **(B, C)**; # means peak anti-Xa 3 h after administration (trough levels were taken max. 2 h before the next dose); x-axis represents days after HIT diagnosis and commencement of fondaparinux treatment.

**Figure 2:**
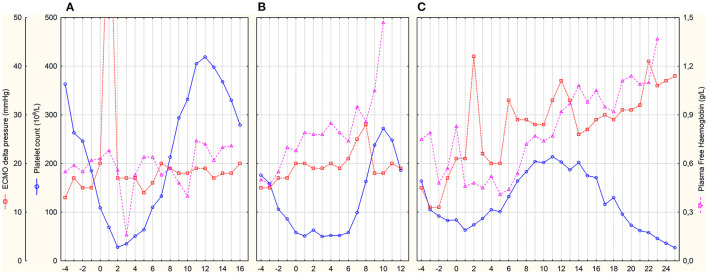
Daily platelet count and markers of ECMO circuit thrombosis. x-axis represents days after HIT diagnosis and commencement of fondaparinux treatment. **(A–C)** represent individual patients.

Patient A was a 63-year-old woman with normal renal function during the entire ICU stay—GFR CKD-EPI was 113 (94–118) ml/min. Fondaparinux was started as a loading dose of 5 mg IV followed by a 2.5 mg IV dose 12-hourly. The determination of anti-Xa by the fondaparinux-calibrated method was not available at that time; therefore, anti-Xa was determined by the LMWH-calibrated assay. Only one anti-Xa value was determined by both methods in parallel (0.48 IU/ml vs. 0.43 mg/L). Fondaparinux doses and anti-Xa levels are shown in Figure 1A. There were several episodes of minor bleeding from the cannulation sites, upper and lower airways, and urinary tract. Fondaparinux doses were intermittently reduced due to bleeding episodes and relatively high anti-Xa levels. No patient-related thrombotic complications were observed. ECMO circuit exchange was necessary 1 day after HIT diagnosis due to significantly elevated ΔP (Figure 2A). Plasma free hemoglobin was moderately elevated throughout the whole course of ECMO therapy (Figure 2A). She underwent a surgical tracheostomy on day 14 of fondaparinux therapy; fondaparinux was withheld for 24 before the procedure. She died after therapy limitation on day 33 of her hospital stay due to persistent severe lung failure.

### 2.2. Patient B

Patient B was a 61-year-old man with initially normal renal function (GFR CKD-EPI 125 (122–135) ml/min until day 9) who developed acute kidney injury on day 9. Continuous veno-venous hemodialysis (CVVHD) was used for the last 3 days of his ICU stay. Fondaparinux loading dose of 5 mg IV was followed by 2.5 mg SC 8-hourly. Trough anti-Xa levels were determined by fondaparinux-calibrated assay (Figure 1B). From day 6, fibrin deposits were visible in the ECMO oxygenator. The ECMO circuit had to be exchanged on day 8 due to impaired ability to eliminate CO_2_. Fondaparinux was reduced on day 8 before surgical tracheostomy on day 9. Subsequently, the patient was complicated with pneumonia with empyema. Besides several episodes of minor bleeding from the cannulation sites and lower respiratory tract, this patient experienced a major bleeding according to the International Society on Thrombosis and Haemostasis definition on day 10 (22). Electrocauterization of the bleeding artery in a pressure sore on the tongue and transfusion of 3 units of packed red blood cells (PRBC) was necessary. Since the source of bleeding was the artery injured by teeth and anti-Xa level was below 0.4 mg/L, we do not consider this bleeding related to the fondaparinux therapy. There were no patient-related thrombotic events. Plasma free hemoglobin levels were persistently elevated with further increase after ECMO set exchange (Figure 2B). The terminal peak in plasma free hemoglobin was not solved due to the decision not to exchange the ECMO circuit because of poor prognosis. The patient died due to septic shock with multiple organ failure after 20 days of ICU stay.

### 2.3. Patient C

Patient C was a 52-year-old man with normal renal function [GFR CKD-EPI 167 (123–185) ml/min] during the entire ICU stay. After HIT diagnosis, fondaparinux was started with a 5 mg IV loading dose followed by 2.5 mg SC 8-hourly. Trough anti-Xa levels were also determined by fondaparinux-calibrated assay (Figure 1C). From day 0 on, blood clots were detected in the oxygenator. Due to an increase in ΔP (Figure 2C) with concomitant deterioration of blood gases, the ECMO circuit was exchanged on day 2. Fondaparinux dose was decreased on day 3 due to the cannulation site and lower respiratory tract bleeding. A thrombotic complication—ischemia of fingers on both hands—was diagnosed on day 6. Although perfusion of the left hand improved later, necrosis of two fingers developed on the right hand. On day 8, fondaparinux was omitted for 24 h, and a surgical tracheostomy was performed on day 9. Subsequently, the inter-dose interval was resumed at 6 h until day 35. On day 15, barotrauma required the insertion of a chest drain with the subsequent complication of chest drain and lower respiratory tract bleeding. Fibrin deposits, detected in the oxygenator on day 22, were accompanied by a mild increase in ΔP and plasma free hemoglobin (Figure 2C). Sepsis with thrombocytopenia gradually progressed while lung function remained stationary poor. The patient died after 36 days of ECMO support and ICU stay.

One episode of major bleeding unrelated to fondaparinux therapy and minor bleedings are described in case descriptions. The administration of PRBC is summarized in Table 1. Neither fresh frozen plasma nor platelets were administered during fondaparinux therapy.

## 3. Discussion

This case series provides a detailed insight into fondaparinux anticoagulation in ECMO patients complicated with HIT, who are prone to develop both bleeding and thrombotic complications, and the anticoagulation therapy becomes exceptionally challenging. In a group of three patients, we proved the fondaparinux efficacy demonstrated by decent ECMO circuit lifespan during the prolonged treatment. One major bleeding episode was unrelated to fondaparinux therapy, and transfusion requirement was low and comparable with a large cohort of veno-venous ECMO patients (23). Daily monitoring and related dosage adjustments contributed to achieving the predefined target plasma concentrations. We believe that this tight control secured the efficacy and safety of fondaparinux therapy.

The majority of previously published cases on fondaparinux anticoagulation on ECMO stated only prophylactic dosage (2.5 mg daily) without an anti-Xa measurement (9, 10). This approach might be insufficient for patients without an increased risk of bleeding because the HIT guidelines recommend using full-dose anticoagulation once the HIT diagnosis has been established (24). ECMO circuits might be extremely prone to clotting due to a hypercoagulable state during HIT and reduced anticoagulation therapy, as demonstrated by the early ECMO circuit exchange in two of three patients. Only one published case report (11) used a therapeutic dosage (7.5 mg daily) with a target anti-Xa range of 0.8–1.2 IU/ml; however, no achieved anti-Xa were reported.

### 3.1. Disadvantages/risks

The major disadvantage of fondaparinux is its long biologic half-life in combination with the unavailability of an antidote. This combination presents a significant risk in case of bleeding because the anticoagulation effect would persist for many hours or days. Moreover, impaired renal functions—a common complication in critically ill patients—further prolong the elimination half-life with a need for dose adjustment and the risk of prolonged bleeding. In contrast to APTT, monitoring of anti-Xa calibrated with fondaparinux is not widely spread. The optimal therapeutic target is unknown; however, this is similar to the other alternative drugs. Generally, fondaparinux is not suitable for patients with a high risk of bleeding and with limited benefits in patients with renal impairment.

### 3.2. Pharmacoeconomics

Although fondaparinux is an off-label drug for patients with HIT, it is an easily available and most affordable option in the Czech Republic. The other options are argatroban and bivalirudin, which have limited availability in some countries. The daily doses of bivalirudin and argatroban were calculated using the previously published dose requirements in ECMO patients (25, 26), and a body weight of 80 kg (see Table 2). Actual prices in the Czech Republic were used except for bivalirudin, which is not available on the Czech market, and where the price from two studies from the USA was adopted (~420 USD per 250 mg vial) (27, 28). Another factor for pharmacoeconomic analysis is the size of the vial, which is usually higher than the required daily dose in the case of argatroban. Once opened, the unused part must be discarded within 24 h. For calculations in Table 2, we used the price of one 100 mg argatroban vial per day. Typical monitoring for argatroban and bivalirudin is APTT 3–4 times daily, while once daily anti-Xa determination seems a reasonable option for fondaparinux due to its long half-life.

**Table 2:** Pharmacoeconomics—expenses per day of treatment, including monitoring.

**Drug**	**Fondaparinux**	**Bivalirudin**	**Argatroban**
Daily dose	7.5 mg	576 mg (0.3 mg/kg/h)	35 mg (0.3 μg/kg/min)
Price of treatment (USD)	16.8	967	308
Monitoring (*n* per day)	Anti-Xa (1)	APTT (3)	APTT (3)
Price of monitoring (USD)	24.8	12.3	12.3
Total price per day (USD)	41.6	979.3	320.3

## 4. Conclusion

In this case series report, all three patients on ECMO received fondaparinux as an alternative anticoagulation agent in HIT. The average time of fondaparinux administration was 18 days. A therapeutic dose given as a loading dose followed by several daily maintenance doses adjusted to reach the target trough anti-Xa of 0.4–0.7 mg/L provided effective, safe, and economically acceptable form of anticoagulation.

## Data availability statement

The original contributions presented in the study are included in the article/supplementary material, further inquiries can be directed to the corresponding author.

## Ethics statement

Ethical review and approval was not required for the study on human participants in accordance with the local legislation and institutional requirements. Written informed consent was obtained from the next of kin to participate in this study. Written informed consent for the publication of this case series was obtained from the next of kin.

## Author contributions

Conceptualization, drafting the manuscript, and collecting data: JR and PS. Critical revision of the manuscript: VŠ. All authors approved the submitted version.
